# A cross-sectional study of avian influenza virus in poultry markets of Guangdong China, 2025

**DOI:** 10.3389/fcimb.2026.1914072

**Published:** 2026-08-11

**Authors:** Wenwen Deng, Silin Xiang, Rongfei Che, Qianfang Guo, Shuling Li, Jiadian Cao, Zhe Liu, Yuxi Yan, Yuwei He, Chuting Zeng, Heshi Long, Aiping Deng, Zhencui Li, Jing Lu

**Affiliations:** 1 School of Public Health, Southern Medical University, Guangzhou, China; 2 Guangdong Provincial Institution of Public Health, Guangdong Provincial Center for Disease Control and Prevention, Guangzhou, China; 3 Guangdong Provincial Key Laboratory of Pathogen Detection for Emerging Infectious Disease Response, Guangdong Workstation for Emerging Infectious Disease Control and Prevention, Guangdong Provincial Center for Disease Control and Prevention, Guangzhou, China; 4 School of Public Health, Guangdong Pharmaceutical University, Guangzhou, China; 5 School of Public Health, Sun Yat-sen University, Guangzhou, China; 6 School of Basic Medicine and Public Health, Jinan University, Guangzhou, China; 7 College of Science, Shantou University, Shantou, China; 8 School of Mathematics and Computational Science, Guilin University of Electronic Technology, Guilin, China

**Keywords:** avian influenza virus, cross-sectional study, genetic diversity, Guangdong, live poultry market

## Abstract

**Background:**

Guangdong, China is a critical epicenter for avian influenza virus (AIV) emergence, where H5N1 and highly pathogenic H7N9 were first identified. Monitoring AIV genotype distribution and genetic diversity in this region is essential for providing early warnings and mitigating the risk of human infections.

**Methods:**

In May 2025, a cross-sectional study was conducted across 4 representative cities in Guangdong. We collected 500 environmental swabs and 45 wastewater samples from 23 live poultry markets (LPMs). Samples were screened for influenza A virus (IAV) and subtyped for H5, H7, and H9. Next generation sequencing (NGS) and phylogenetic analyses were employed to characterize viral diversity and zoonotic potential.

**Results:**

AIV was detected in 49.2% (246/500) of environmental samples. H9 was the dominant subtype (38.2%), followed by H5 (3.2%) and H7 (0.8%); 2.4% of positive samples showed multi-subtype co-occurrence. “Non-restricted zone” markets (80.0%) and “waste storage areas” (68.4%) were identified as primary contamination hotspots. Phylogenetic analysis revealed that H5 and H9 viruses are accumulating mutations linked to enhanced human-type receptor affinity. Notably, H5 and H6 sequences exhibited long internal branches and clustered with isolates from other Asian countries rather than domestic strains, highlighting a significant gap in current domestic molecular surveillance and a lack of continuous evolutionary data for these genotypes.

**Conclusions:**

Widespread AIV contamination persists in Guangdong LPMs, dominated by H9 with low-level circulation of highly pathogenic subtypes. The identified surveillance gaps for H5/H6 and the presence of multi-subtype environments underscore the urgent need for strengthened, integrated molecular monitoring and stricter market regulation to mitigate public health risks.

## 1 Introduction

Avian Influenza Virus (AIV), naturally occurring in wild aquatic birds, represent a persistent threat to global poultry production and public health. The segmented RNA genome of these viruses facilitates genetic reassortment, a mechanism that drives rapid evolution and the emergence of novel strains, particularly when different subtypes co-circulate in poultry (Su et al., 2021; Jakob et al., 2022; Sharma et al., 2024). In recent years, the global AIV epidemic has been characterized by the co-circulation of multiple subtypes, frequent cross-host infections, and an increase in gene reassortment events, with the H5, H7, and H9 subtypes being of greatest concern. The H5 subtypes, particularly clade 2.3.4.4b H5N1, have caused unprecedented panzootics in wild birds and poultry across Asia, Europe, Africa, and the Americas since 2020 (De Marco et al., 2023; Tian et al., 2023). Continuing into 2024, these viruses have demonstrated an alarming capacity for cross-species transmission, leading to massive outbreaks in mammals (Sreenivasan et al., 2024) and heightening pandemic concern. In contrast, the H7N9 subtype, responsible for five waves of human infections in China from 2013 to 2017, has been virtually eliminated from poultry populations following extensive national vaccination programs (Li and Chen, 2021; Guinat et al., 2023), though sporadic environmental detections warrant continued vigilance. Meanwhile, the low-pathogenicity H9N2 subtype remains enzootic in poultry across vast regions of Asia and Africa (Carnaccini and Perez, 2020; Fusaro et al., 2024). Its high prevalence enables it to frequently act as a “gene donor,” providing internal gene segments to other AIVs, thereby posing a persistent risk for generating novel reassortant viruses with zoonotic potential, with occasional human cases being reported (Bao et al., 2022; Fan et al., 2024).

The existing human epidemic history of both H5N1 and H7N9 indicates that live poultry markets have been unequivocally identified as critical nexuses for the amplification, interspecies transmission, and genetic diversification of AIVs (Yuan et al., 2016; Wang et al., 2023). By congregating poultry from diverse geographic and farm-system origins, live poultry markets (LPMs) create high-density environments conducive to viral shedding and co-occurrence. This setting not only facilitates reassortment events but also serves as a primary interface for human exposure (Naguib et al., 2021; Hassan et al., 2023), particularly in regions with strong cultural preferences for fresh poultry. Consequently, systematic surveillance within LPMs is an indispensable tool for monitoring AIV genetic diversity, tracking epidemiological shifts, and assessing the risk of zoonotic emergence in real-time.

Guangdong province in southern China plays a pivotal role in the global ecology of AIV transmission and evolution. This high-risk status is driven by a confluence of factors: a dense human population, a subtropical climate favorable for viral persistence, vast and interconnected poultry farming and trade networks (Liu et al., 2020; Sun et al., 2023), and deep-rooted dietary habits favoring freshly slaughtered poultry from LPMs. Historically, Guangdong has served as a critical sentinel region, being the site where the highly pathogenic H5N1 progenitor—A/goose/Guangdong/1/1996 (Gs/GD)—and emergent highly pathogenic H7N9 variants were first identified (Zhu et al., 2025; Kang et al., 2017). Therefore, conducting AIV surveillance in Guangdong is of paramount representative significance for understanding regional viral dynamics and assessing global pandemic risk.

In this study, we conducted comprehensive cross-sectional investigation in LPMs across representative cities in the eastern, western, southern, and northern regions of Guangdong. Our objective was to characterize the current prevalence, subtype distribution, and molecular evolutionary features of AIVs circulating in this key region. The findings will provide a crucial evidence-based foundation for refining and optimizing avian influenza prevention and control strategies.

## 2 Methods

### 2.1 Monitoring site selection

In May 2025, this study selected four representative cities—Dongguan (Pearl River Delta region in center of Guangdong), Meizhou (Eastern Guangdong), Maoming (Western Guangdong), and Qingyuan (Northern Guangdong)—as monitoring points. In each city, 5–6 sampling sites were randomly chosen, covering five types of venues: poultry slaughterhouse, live poultry wholesale market, live poultry retail market, market in restricted zone, and live poultry market in non-restricted zone. In this context, “restricted zones” refer to areas where live poultry trading has been banned by public health authorities as a control measure, while “non-restricted zones” are areas without such a ban.

### 2.2 Sample collection

Samples were collected via two approaches at each site. First, environmental samples were gathered using sterile swabs from high-contact surfaces, including cage surfaces, chopping blocks, poultry feces, washing water, knives, and tabletops. Each swab was placed into an individual collection tube, and no sample pooling was performed at any stage. Second, a volume of 250 mL of wastewater was concurrently collected from the market drainage outlets per sample in sterile bottles. No transport media or enrichment culture media were added to the wastewater samples during collection or transportation. All samples were immediately stored at 4 °C post-collection and transported to the laboratory via cold chain within 24 hours for nucleic acid detection.

### 2.3 Nucleic acid extraction

Viral nucleic acids were extracted from all collected samples, with distinct pretreatment protocols for environmental and wastewater samples. For environmental samples, 200 µL of the sample was directly added to the lysis buffer and processed for nucleic acid extraction using a Nucleic Acid Extraction Kit (Magen, Guangzhou, China). The final nucleic acid product was eluted in 80 µL of elution buffer.

For wastewater samples, a 15 mL aliquot was mixed with 300 µL of a lysis-concentration solution and incubated at room temperature for 5 minutes for lysis. The mixture was then centrifuged at 2,000 × g for 1 minute to remove particulate matter. Subsequently, viral nucleic acids were extracted from 10 mL of the resulting supernatant using a magnetic bead-based Large Volume Water Viral DNA/RNA Extraction Kit (BayBiopure, Guangzhou, China). The final nucleic acids were eluted in 80 µL of elution buffer.

All extracted nucleic acids were then used as templates for subsequent real-time reverse transcription PCR (RT-qPCR) and next generation sequencing (NGS) analysis.

### 2.4 RT-qPCR detection

All samples were initially screened for Influenza A Virus (IAV) by a real-time reverse transcription PCR assay using universal primers and probes. IAV-positive samples were subsequently subtyped for H5, H7, and H9 using a commercial RT-qPCR kit (Avian Influenza Virus H5/H7/H9 Subtype Nucleic Acid Detection Kit, Berger, Shanghai, China). All primers and probes for the universal IAV and subtyping assays were those recommended by the Chinese National Influenza Center (https://ivdc.chinacdc.cn/cnic/) in its standard operating procedures (Chinese National Influenza Center, China CDC, 2012).

### 2.5 Virus isolation

Environmental samples that tested positive for IAV with a cycle threshold value of ≤ 32 were selected for virus isolation attempts. Selected samples were inoculated into the allantoic cavity of 9- to 11-day-old specific-pathogen-free (SPF) embryonated chicken eggs (Bo et al., 2025). Virus isolation was not attempted for the wastewater samples.

### 2.6 Next generation sequencing

For environmental samples yielding successful virus isolation, whole-genome sequencing (WGS) of the isolates was performed by Geneplus Co., Ltd. (Beijing, China). For environmental samples without isolates and all raw sewage samples, targeted next-generation sequencing (tNGS) was conducted (Li et al., 2026). Total nucleic acids were extracted in-house and submitted to KingCreate Biotechnology (Guangzhou, China) for sequencing library preparation using the MetaVirus hybridization capture reagents and accompanying kits (KS668-MetaV-12). Viral nucleic acids were first reverse-transcribed into cDNA and then fragmented into 200–500 bp segments. These segments were subsequently ligated with barcode-indexed adapters to construct the sample nucleic acid sub-libraries. To enhance the detection sensitivity, target viral sequences were enriched via hybridization capture using a massive broad-spectrum probe panel covering over 1,300 viruses and more than 15,000 viral strains. Finally, the captured libraries were subjected to high-throughput sequencing to obtain raw sequencing data.

### 2.7 Data analysis

#### 2.7.1 Statistical analysis

Data were collated using Microsoft Excel and statistically analyzed with SPSS version 27.0.1. Figures were generated using R Studio and Adobe Illustrator 2025. The chi-square (*χ²*) test was used to compare positive rates between different groups. A *p*-value of less than 0.05 was considered statistically significant.

#### 2.7.2 Phylogenetic analysis

Raw sequencing data first underwent quality control, filtering, trimming, and adapter removal using Fastp (Chen, 2023). Subsequently, bioinformatics analysis was performed using the EsViritu pipeline (v0.2.3) (https://github.com/cmmr/EsViritu) (Tisza et al., 2023). Qualitative identification of viral species was achieved by aligning the processed sequencing reads against a reference genome database, while viral abundance in the samples was calculated concurrently. To elucidate the epidemiological characteristics and evolutionary dynamics of AIVs in Guangdong Province, our resulting genomic data were integrated with recent hemagglutinin (HA) sequences of prevalent global H5, H6, H7, and H9 strains retrieved from public databases, including GISAID (https://gisaid.org/) and NCBI (https://www.ncbi.nlm.nih.gov/), for a comprehensive phylogenetic analysis. A maximum likelihood phylogenetic tree was constructed using FastTreeMP (Vale et al., 2021) under the General Time Reversible (GTR) model with 1000 bootstrap replicates. The results were visualized using Auspice (https://auspice.us/) (Hadfield et al., 2018), the web-based visualization tool of the Nextstrain platform.

## 3 Results

### 3.1 Detection of avian influenza virus in LPM environmental samples

During May 2025, a cross-sectional environmental survey was conducted in LPMs across four typical regions of Guangdong Province (Pearl River Delta region, Eastern, Western, and Northern Guangdong), with a total of 500 environmental samples collected. 246 samples (49.20%) were detected as IAV positive.

**Figure 1:**
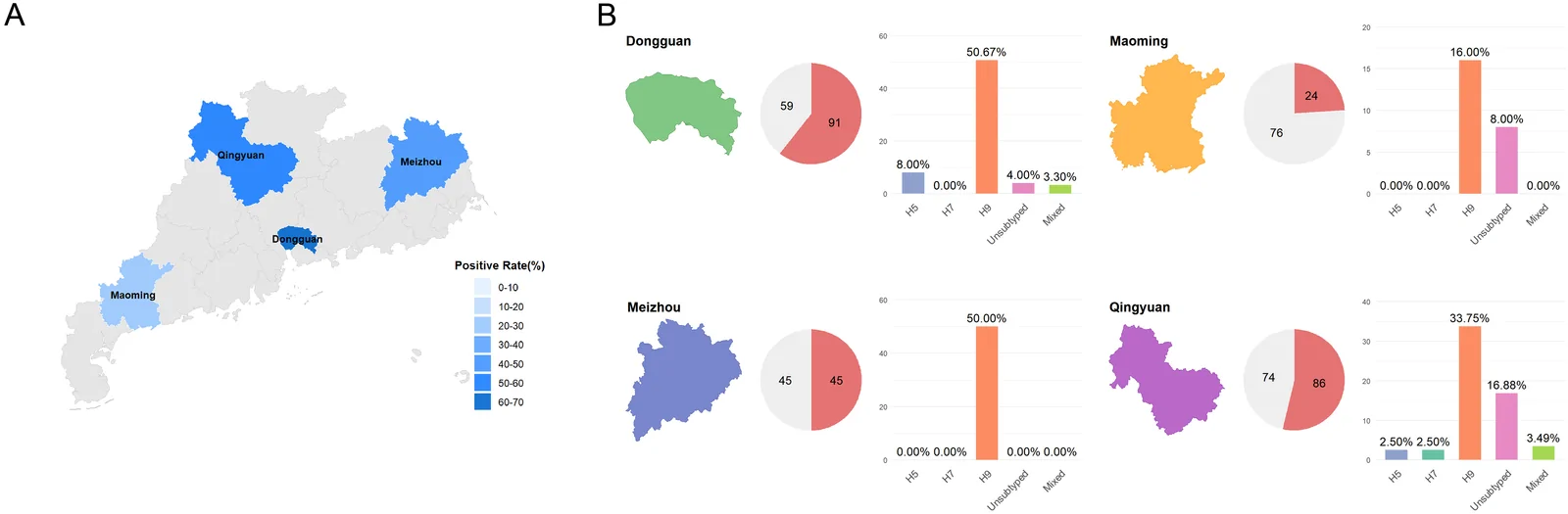
Prevalence of AIVs in LPMs of Guangdong Province, May 2025. **(A)** Geographical distribution of the surveyed LPMs. The four studied cities are color-coded in shades of blue, with the color intensity corresponding to the AIV positive detection rate in environmental samples (darker shades indicate higher rates). **(B)** AIV detection in environmental samples from the four cities. The pie charts illustrate the proportion of AIV-positive (red) and AIV-negative (gray) environmental samples for each city, with the numbers in black indicating the sample count for each category. The bar chart shows the positive detection rates of different AIV subtypes. The “Mixed rate” represents the proportion of AIV co-occurrence samples among all positive samples within that city.

For regional distribution, Dongguan in Pearl River Delta region with the highest population density presented the highest IAV positive rate (91/150, 60.67%), followed by Qingyuan (86/160, 53.75%) and Meizhou (45/90, 50.00%). The LPM in western region of Guangdong (Maoming) had the lowest positive rate (24/100, 24.00%). The differences in AIV positive rates among the regions were statistically significant (*χ²* = 34.648, *p* < 0.001) (**Figure 1A**).

### 3.2 Virus subtype distribution in LPMs

AIV positive samples were subtyped for H5, H7, and H9 by RT-PCR. Samples positive for IAV but negative for H5/H7/H9 were categorized as “other” subtypes. The results showed that the H9 subtype had the highest detection rate (191/500, 38.20%), followed by the H5 subtype (16/500, 3.20%) and the H7 subtype (4/500, 0.80%). There were also 41 other subtype samples (8.20%). 6 of 246 (2.44%) AIV positive samples contained both H5 and H9 subtypes.

While H9 was the predominant subtype across all sites, distribution patterns varied significantly by city (**Figure 1B**), likely reflecting distinct regional live poultry trading routes. In Dongguan and Qingyuan, viral profiles were more diverse; Dongguan reported 80.22% H9 and 9.89% H5, while Qingyuan showed a mix of H9 (59.30%), H7 (4.65%), and H5 (1.16%). Both cities also identified H5/H9 co-occurrence (3.30% and 3.49%, respectively), posing a high risk for genetic reassortment. Conversely, subtype diversity was lower in Meizhou, where only H9 was detected (100%), and Maoming, which lacked H5, H7, or co-occurrence despite H9 dominance (66.67%). These spatial variations suggest that localized trade networks influence the circulation and complexity of AIV genotypes within the province.

### 3.3 AIVs distribution by sample and market types

For risk assessment, we compared the AIV positive rates among different type of samples collected from LPMs. The relative high positive rate were found in poultry drinking water (28/54, 51.85%), swabs from poultry slaughtering or display chopping blocks (55/107, 51.40%) and “other” samples (107/207, 51.69%) which primarily included those collected from feeding troughs, slaughtering knives, trays, floors, freezers, and trash cans. The positive rates for fecal samples and cage surface swabs were 45.31% (29/64) and 39.71% (27/68), respectively (**Table 1**).

**Table 1:** Avian influenza nucleic acid detection results of different types of environmental samples in four cities of Guangdong, May 2025.

Sample type	Positive rates of each subtype								Mixed positive	
	H5		H7		H9		Unsubtyped			
	n	%	n	%	n	%	n	%	n	%
Slaughtering/Chopping Block	6	5.61	2	1.87	43	40.19	8	7.48	4	7.27
Cage Surface Swab	1	1.47	0	0.00	22	32.35	4	5.88	0	0.00
Poultry Drinking Water	0	0.00	0	0.00	26	48.15	2	3.70	0	0.00
Fecal Sample	0	0.00	1	1.56	23	35.94	5	7.81	0	0.00
Other	9	4.35	1	0.48	77	37.20	22	10.63	2	1.87
Total	16	3.20	4	0.80	191	38.20	41	8.20	6	2.44

The counts for individual subtypes and mixed positive samples are not mutually exclusive. Additionally, the percentage of “Mixed Positive” represents the proportion of mixed samples out of the AIV-positive samples within that specific category.

**Figure 2:**
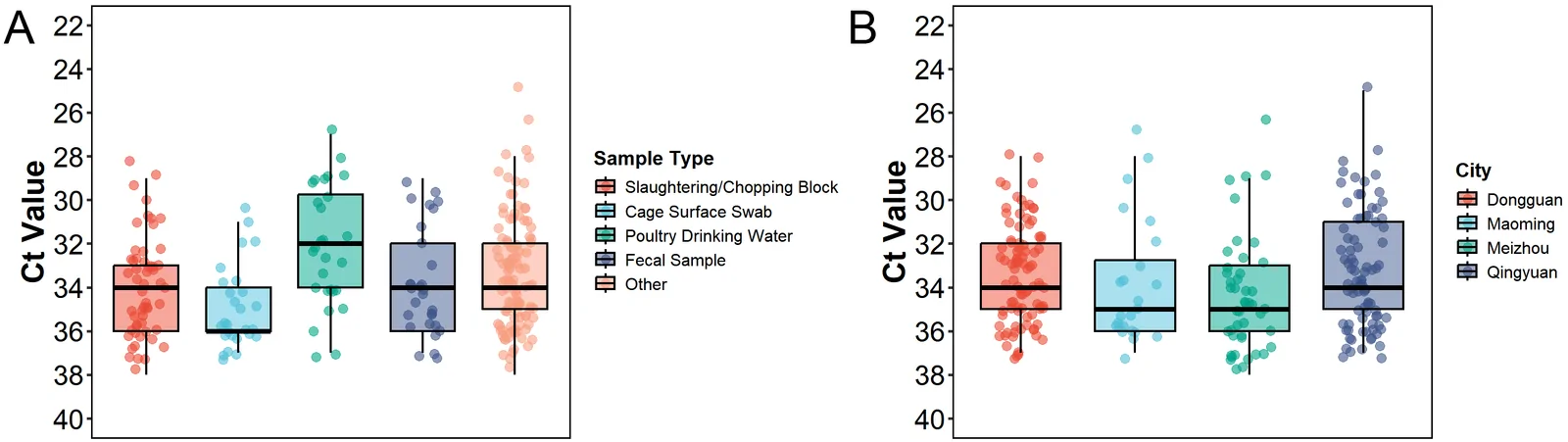
Distribution of viral load, represented by cycle threshold (Ct) values, in AIV-positive environmental samples. The boxplots compare the distribution of Ct values across different sample types and cities, where lower Ct values indicate higher viral loads.In each boxplot, the central line represents the median; the box boundaries indicate the 25th and 75th percentiles (interquartile range, IQR); and the whiskers extend to 1.5 times the IQR from the box boundaries. Data points beyond the whiskers are plotted as individual outliers. **(A)** Distribution of Ct values among different environmental sample types. **(B)** Distribution of Ct values among the four surveyed cities.

Significant differences in viral loads were identified for different sample types (*p* < 0.001) and also location sites (*p* < 0.05), suggesting that both sample type and location are key factors influencing the level of environmental viral contamination (**Figure 2**).

The environmental samples collected in this study covered five types of poultry-related venues: poultry slaughterhouse, live poultry wholesale market, live poultry retail market, market in restricted zone (i.e., where live poultry sales were banned), and live poultry market in non-restricted zone. The most severe AIV contamination was identified in non-restricted zone LPM, which exhibited the highest positive rate for influenza A virus at 80% (16/20). This was followed by restricted zone market (98/150, 65.33%), live poultry retail market (62/120, 51.67%), and live poultry wholesale market (66/170, 38.82%). Poultry slaughterhouse had the lowest AIV positive rate (10.00%, 4/40). Statistical analysis revealed a significant difference in the avian influenza virus positive rates among the various poultry trading venues (*χ²* = 55.420, *p* < 0.001) (**Table 2**).

**Table 2:** Nucleic acid detection results of environmental samples from different poultry-related sites in four cities of Guangdong, May 2025.

Premise type	Positive rates of each subtype								Mixed positive	
	H5		H7		H9		Unsubtyped			
	n	%	n	%	n	%	n	%	n	%
Poultry Slaughterhouse	0	0.00	0	0.00	2	5.00	2	5.00	0	0.00
Wholesale LPM	0	0.00	4	2.35	49	28.82	13	7.65	0	0.00
Retail LPM	1	0.83	0	0.00	51	42.50	10	8.33	0	0.00
Restricted Zone Market	15	10.00	0	0.00	73	48.67	16	10.67	6	6.12
Non-restricted Zone LPM	0	0.00	0	0.00	16	80.00	0	0.00	0	0.00
Total	16	3.20	4	0.80	191	38.20	41	8.20	6	2.44

The counts for individual subtypes and mixed positive samples are not mutually exclusive. Additionally, the percentage of “Mixed Positive” represents the proportion of mixed samples out of the AIV-positive samples within that specific category.

**Figure 3:**
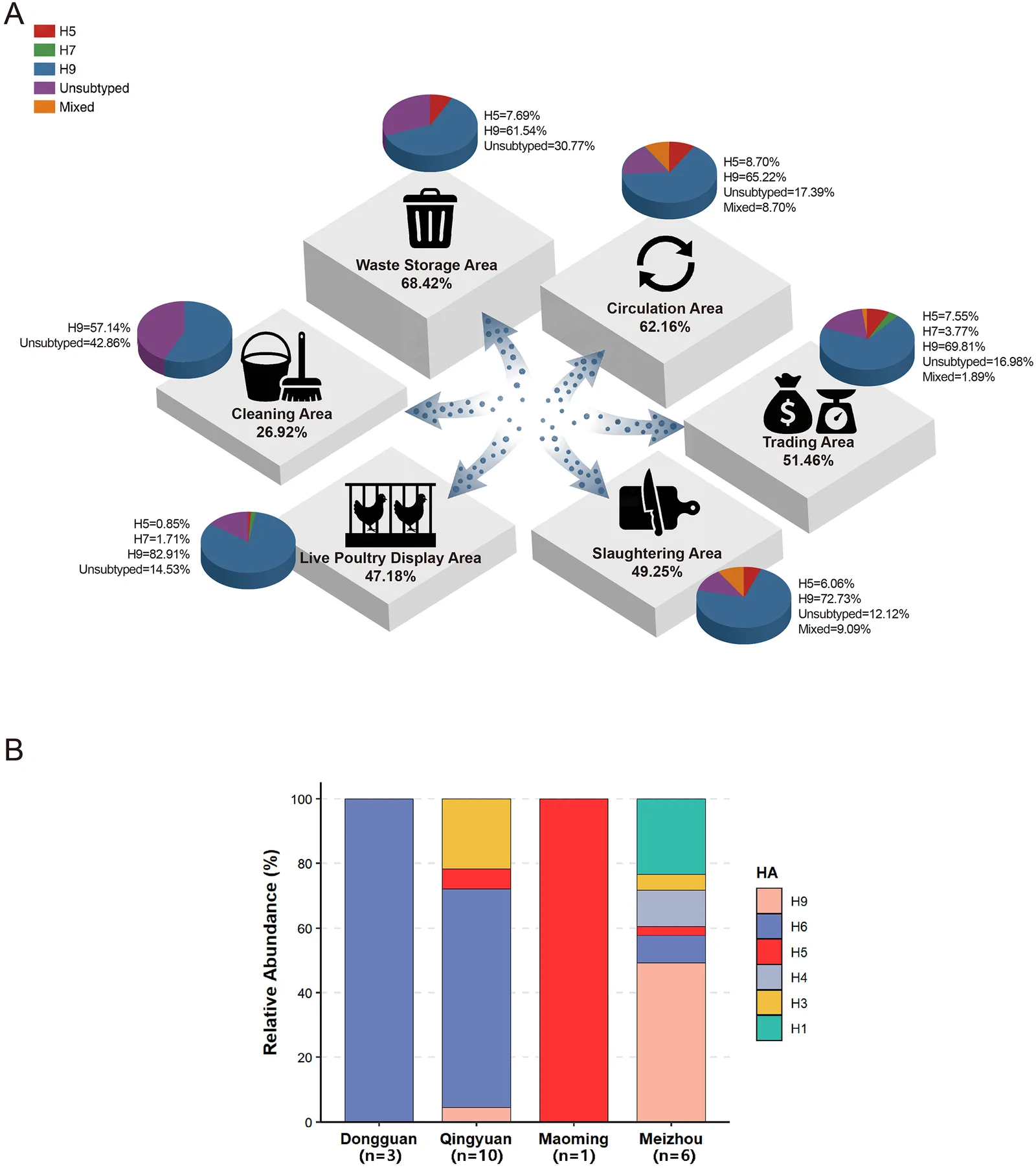
Prevalence and subtype distribution of AIV in environmental and wastewater samples from Guangdong live poultry markets, May 2025. **(A)** AIV prevalence and subtype distribution in environmental swab samples collected from different functional areas of live poultry market stalls. The percentage below each icon indicates the overall AIV positive rate for that specific area. The corresponding pie charts illustrate the subtype composition of the AIV-positive samples. **(B)** Relative abundance of AIV hemagglutinin subtypes in wastewater samples from the four representative cities, identified by targeted next-generation sequencing. The stacked bar chart displays the percentage composition of different HA subtypes. The number of samples (n) for each city is indicated on the x-axis.

Based on the actual layout of the monitored market stalls, an individual poultry stall typically consists of six functional areas: live poultry display area, slaughtering and processing area, trading area, cleaning area, waste storage area, and circulation area. The waste storage area had the highest AIV positive rate (13/19, 68.42%), followed by the circulation area (23/37, 62.16%). The positive rates in the trading area (53/103, 51.46%), slaughtering and processing area (33/67, 49.25%), and live poultry display area (117/248, 47.18%) were close to the overall market average (49.20%). The cleaning area had the lowest positive rate (7/26, 26.92%). These data indicate a statistically significant difference in AIV positive rates across the market’s functional areas (*χ²* = 11.074, *p* = 0.050) (**Figure 3A**).

### 3.4 AIV distribution in LPM wastewater

The wastewater surveillance serve as a non-invasive and systematic reflection of the overall health status of the covered population (Li et al., 2024), and could also serve as an efficient tool to monitor the AIVs circulation in LPMs. We analyzed 45 wastewater samples collected from drainage outlets across four study sites using targeted next-generation sequencing. To accurately quantify viral loads, the relative abundance of IAV was calculated based on Reads Per Kilobase per Million mapped reads (RPKM), a normalization method that corrects for variations in gene length and sequencing depth. The analysis showed that IAV accounted for an average of 20.60% of the total viral community in wastewater, but with significant regional differences. Qingyuan had the highest IAV abundance (60.10%), followed by Meizhou (8.95%), while Dongguan (2.09%) and Maoming (0.71%) had lower levels.

Notably, the hemagglutinin subtype composition in wastewater displayed marked geographical variation (**Figure 3B**). Unlike the widespread dominance of H9 in environmental swabs, wastewater profiles were distinct by city. Dongguan and Maoming showed single-subtype dominance, comprising 100% H6 and 100% H5, respectively. Qingyuan was dominated by H6 (67.69%) and H3 (21.70%), with minor detections of H5 and H9. Conversely, Meizhou exhibited the greatest diversity, characterized by a predominance of H9 (49.18%) and a unique, significant presence of H1 (23.40%) and H4 (11.29%), alongside H3, H5, and H6. Concurrently, analysis of neuraminidase subtypes identified N2 and N6 as the most prevalent types, with N1 also being detected, presented in **Supplementary Figure 1**. These findings highlight that wastewater surveillance can capture a broader spectrum of circulating HA/NA subtypes, such as H1, H3, and H6, which may be underestimated by conventional swab-based sampling.

### 3.5 Phylogenetic and molecular characterization of AIVs

**Figure 4:**
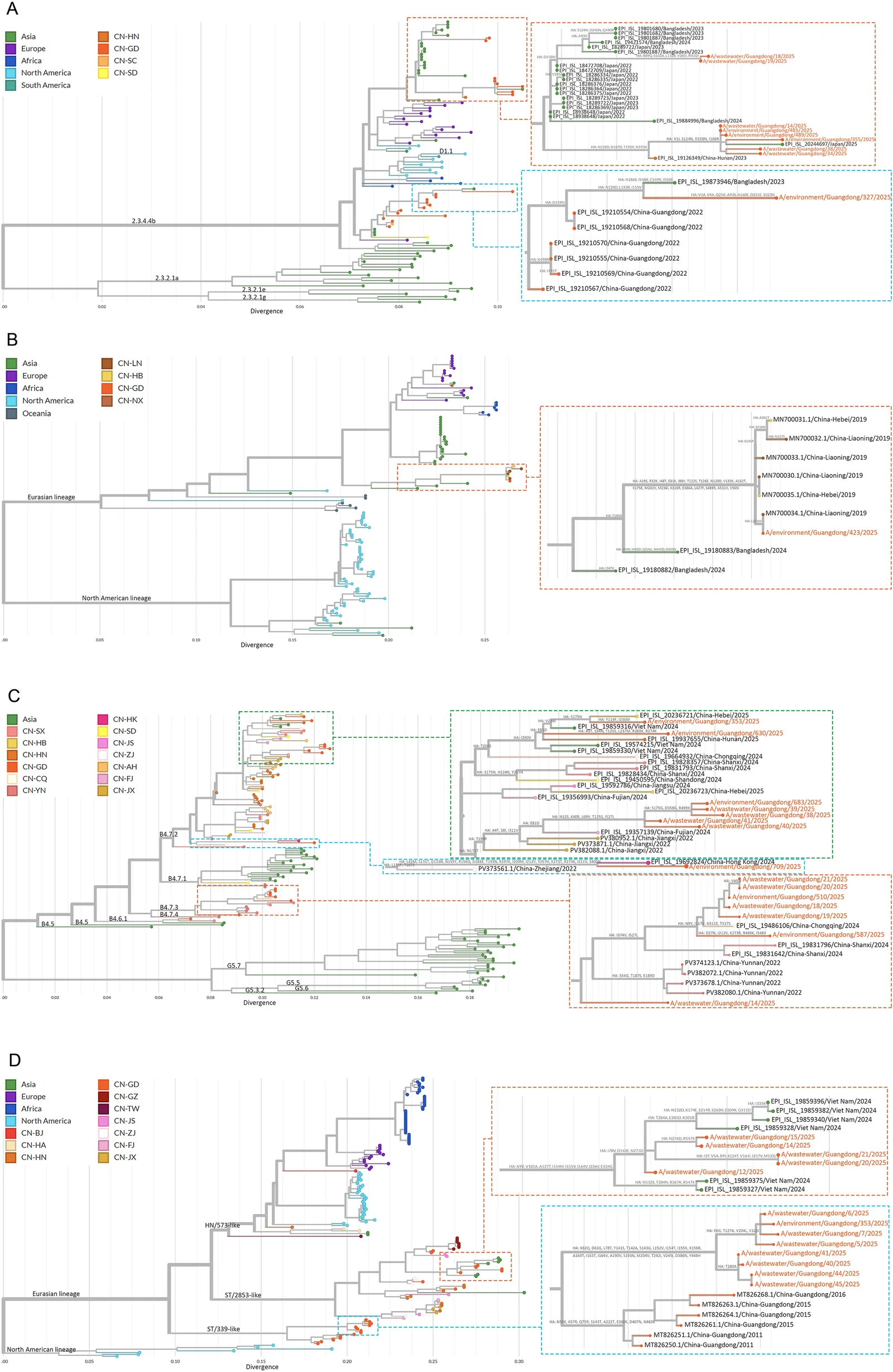
Phylogenetic analysis of the hemagglutinin genes of AIVs identified in live poultry markets of Guangdong Province. Maximum likelihood (ML) phylogenetic trees were constructed for the HA genes of **(A)** H5, **(B)** H7, **(C)** H9, and **(D)** H6 subtypes, illustrating the evolutionary context of the AIV HA gene sequences obtained in this study. Left panel: The phylogenetic distance tree, where branch lengths are proportional to the number of nucleotide substitutions per site, highlighting the genetic divergence among the viruses. Right panel: A magnified view of the specific clade that contains the study sequences, revealing their detailed phylogenetic topology and relationships with their closest genetic relatives. To ensure accurate and consistent alignment, all amino acid positions were described according to the H3 numbering system.

To systematically characterize the evolutionary features of AIVs circulating in Guangdong live poultry markets, we constructed Maximum Likelihood phylogenetic trees for the H5, H6, H7, and H9 subtypes based on 38 hemagglutinin (HA) gene sequences obtained from environmental and wastewater samples (**Figure 4**).

A total of nine H5 sequences were identified, all belonging to the globally dominant clade 2.3.4.4b (**Figure 4A**). Notably, these sequences did not cluster closely with recent domestic isolates; instead, they formed distinct phylogenetic groups with sequences from Japan and Bangladesh. The presence of long internal branches in the H5 tree (**Figure 4A**, left panel) suggests a significant spatio-temporal gap in sequence data, likely indicating a lack of systematic molecular surveillance for this genotype within the region. This sequence scarcity limits our ability to describe the continuous evolutionary trajectory of H5 viruses domestically. Molecular characterization revealed several mutations at the receptor-binding site (RBS) and antigenic regions. Key substitutions in the 190-helix (T192K/I, N193K) and the 130-loop (N128D, L133R) were observed. Furthermore, the I166R mutation in antigenic site B was identified in six sequences, potentially altering the virus’s antigenicity and zoonotic potential.

In contrast to the international clustering of H5, the H7 and H9 sequences showed high homology with domestic isolates. The identified H7 sequence clustered within the Eurasian lineage, showing 92.9% nucleotide identity with a 2019 Liaoning isolate (**Figure 4B**). As the predominant subtype, all H9 sequences were assigned to the BJ/94-like lineage, specifically sub-lineages B4.7.2 and B4.7.3 (**Figure 4C**). These sequences were closely related to isolates from various Chinese provinces, including Hebei, Hunan, Fujian, and Chongqing. This close phylogenetic proximity to other domestic strains suggests that H7 and H9 lineages are well-characterized and persistently circulating within the established domestic poultry trade networks. All H9 sequences maintained the L226 mutation (H3 numbering), signaling a stable affinity for human-type α-2,6-SA receptors (Bao et al., 2022).

Wastewater metagenomics revealed high H6 abundance, with sequences forming two independent clusters: ST/339-like and ST/2853-like (**Figure 4D**). Similar to H5, the H6 sequences clustered predominantly with isolates from Southeast Asia (e.g., Vietnam) rather than domestic poultry samples. The significant divergence and extended branch lengths observed in **Figure 4D** (left panel) highlight a critical surveillance blind spot for H6 subtypes in China. The lack of contemporary domestic reference sequences suggests that H6 viruses may be circulating and evolving in aquatic poultry or specific market niches, necessitating more robust and continuous molecular monitoring.

## 4 Discussion

Guangdong is a key endemic region for AIV with global significance in its transmission and evolution (Lemey et al., 2009; Lu et al., 2018). However, there has been a notable gap in continuous, comprehensive AIV surveillance data in this region in recent years. To address this and update our understanding of the current epidemiological landscape, this study was designed to answer three specific scientific questions: (1) Which geographic regions within Guangdong currently represent high-risk areas for AIV contamination? (2) How does the viral contamination vary across different market types and specific functional locations within stalls? and (3) What are the current distribution characteristics and molecular evolutionary features of various AIV subtypes? Our cross-sectional survey confirms high environmental AIV contamination (49.2%) in Guangdong live poultry markets, with H9 remaining the dominant lineage. These findings provide a critical, timely update on viral prevalence and genetic evolution despite existing control measures.

The deep enzooticity of H9 subtype in Guangdong, reflecting its firm establishment in local poultry populations, is highly consistent with findings from numerous regions across China and elsewhere in Asia (Gu et al., 2017; Fusaro et al., 2024). Beyond its direct zoonotic threat, H9’s role as a ubiquitous internal gene donor is concerning. The co-detection of H5 and H9 subtypes in single environmental samples from Dongguan and Qingyuan highlights the presence of ecological mixing vessels. This spatial proximity of diverse viral lineages increases the probability of genetic exchange and the potential emergence of novel reassortants.

The observed geographical variation in AIV prevalence likely reflects differences in regional poultry supply chains and vaccination coverage (Bo et al., 2025). The significantly higher contamination in “non-restricted zone” markets validates the efficacy of trade restrictions. In reality, due to strong consumer preference for freshly slaughtered poultry in southern China, many markets continue to sell live birds and offer on-site slaughtering services, which is a major contributing factor to the persistently high levels of AIV contamination over the years. Furthermore, identifying “waste storage areas” as primary hotspots emphasizes that standardized waste management and the rigorous implementation of the “1110” strategy (daily cleaning, weekly disinfection, monthly closure, and zero overnight live poultry) remain essential to breaking the transmission chain.

While environmental swabs provide spatial snapshots, wastewater surveillance offers a panoramic assessment of the market virome. Our tNGS analysis revealed significant circulation of H6 and H3 subtypes, lineages frequently under-detected in routine surveillance programs that prioritize H5, H7, and H9 PCR screening. The predominance of H6 in wastewater may be attributed to its efficient shedding in aquatic poultry excreta and superior stability in aqueous environments compared to other subtypes (Bi et al., 2020). These findings demonstrate that integrating wastewater monitoring and NGS method into existing frameworks provides sensitivity for identifying cryptic circulation, multi-subtype co-occurrence, and low-abundance lineages that otherwise remain “hidden” in surface-based sampling. Molecular characterization further elucidates the complex evolutionary landscape and associated public health risks. A critical finding in our phylogenetic analysis is the presence of significant spatio-temporal gaps in the domestic molecular record. The HA sequences of H5 and H6 subtypes identified in this study exhibited long internal branches and formed distinct clusters with international isolates from Japan, Bangladesh, and Southeast Asia, rather than contemporary domestic strains. We analyzed the spatiotemporal coverage of sequences deposited in the GISAID database between January 1, 2020, and December 31, 2025. During this period, 39,313 H5 and 1,085 H6 sequences were submitted worldwide. Of these, 532 H5 sequences (1.4%) and 65 H6 sequences (6.0%) originated from China. We consider that this persistent under-representation in public databases limits the phylogenetic accuracy of China local lineages. Consequently, the international clustering patterns observed in our study is likely a result of sequence scarcity.

In contrast, H7 and H9 sequences showed high homology with isolates from other Chinese provinces, indicating a more stable and well-characterized domestic circulation within terrestrial poultry trade networks. Nevertheless, the identified H9N2 viruses consistently carried the HA-L226 mutation, signifying a preserved affinity for human-type α-2,6-SA receptors. Coupled with the detection of H5 clade 2.3.4.4b—a lineage with documented mammalian tropism—and specific mutations such as T192K (H5) and N155Y/T193N (H9), our results underscore ongoing viral adaptation for immune evasion and potential cross-species transmission (Xue et al., 2025).

### 4.1 Limitations

There are several limitations of this study. Although this surveillance covered representative cities across four distinct regions of Guangdong, the sampling was conducted exclusively at a single time point in May 2025. Avian influenza viruses are known to exhibit distinct seasonal dynamics, with prevalence and subtype distribution often shifting in response to climatic changes, migratory bird patterns, and seasonal peaks in poultry consumption. Therefore, this cross-sectional snapshot limits our ability to assess the temporal dynamics or seasonal peaks of AIV circulation. Future studies should incorporate longitudinal sampling across multiple seasons to provide a more comprehensive spatiotemporal understanding of AIV ecology in southern China.

In current study, we relied solely on molecular detection without attempting virus isolation from the sewage samples. Consequently, we cannot definitively determine whether the detected broad subtypes in wastewater represent viable, infectious viruses or merely residual nucleic acid fragments. Viral enrichment and live-virus isolation methods should be further optimized for wastewater samples, thereby enabling more accurate assessments of environmental infection risks through LPM-based wastewater surveillance.

## 5 Conclusions

This cross-sectional study highlights the widespread and persistent contamination of avian influenza viruses, predominantly the H9 subtype, in live poultry markets across Guangdong Province. The identification of multi-subtype co-occurrence (e.g., H5/H9) and the significant viral load in “non-restricted zones” and “waste storage areas” underscore these environments as critical interfaces for viral reassortment and zoonotic transmission. Furthermore, the detection of long internal branches in the phylogenetic trees of H5 and H6 subtypes reveals a significant spatiotemporal gap in domestic molecular surveillance. For future directions, it is imperative to implement continuous, longitudinal surveillance spanning multiple seasons to capture the spatiotemporal dynamics of AIVs. Additionally, establishing a comprehensive surveillance system that integrates environmental swab testing with wastewater monitoring will be crucial for capturing hidden viral diversity. Upgrading market hygiene regulations and promoting the centralized slaughtering system will remain essential interventions to mitigate the future risk of novel AIV emergence and safeguard public health.

## Data Availability

The datasets presented in this study can be found in online repositories. The names of the repository/repositories and accession number(s) can be found below: https://ngdc.cncb.ac.cn/gsa/browse/CRA036371, PRJCA054853.

## References

[B1] Bao P. Liu Y. Zhang X. Fan H. Zhao J. Mu M. . (2022). Human infection with a reassortment avian influenza a H3N8 virus: An epidemiological investigation study. Nat. Commun. 13, 6817. doi: 10.1038/s41467-022-34601-1 36357398 PMC9649012

[B2] Bi Y. Li J. Li S. Fu G. Jin T. Zhang C. . (2020). Dominant subtype switch in avian influenza viruses during 2016-2019 in China. Nat. Commun. 11, 5909. doi: 10.1038/s41467-020-19671-3 33219213 PMC7679419

[B3] Bo H. Zhang Y. Dong J. Li X. Zhao X. Wei H. . (2025). Characterization of the avian influenza viruses distribution in the environment of live poultry market in China 2019–2023. Infect. Dis. Poverty 14, 36. doi: 10.1186/s40249-025-01304-w 40346680 PMC12063257

[B4] Carnaccini S. Perez D. R. (2020). H9 influenza viruses: An emerging challenge. Cold Spring Harb. Perspect. Med. 10, a038588. doi: 10.1101/cshperspect.a038588 31871234 PMC7263090

[B5] Chen S. (2023). Ultrafast one‐pass FASTQ data preprocessing, quality control, and deduplication using fastp. Imeta 2, e107. doi: 10.1002/imt2.107 38868435 PMC10989850

[B6] Chinese National Influenza Center, China CDC . (2012). Standard Operating Procedures of the Chinese National Influenza Center. Available online at: https://ivdc.Chinacdc.cn/sysgl/swaq/jszl/201203/P020120323392850626391.pdf (Accessed January 7, 2026).

[B7] De Marco M. A. Delogu M. Cotti C. (2023). Special issue “ecology of influenza a viruses”: Editorial. Microorganisms 11, 1287. doi: 10.3390/microorganisms11051287 37317261 PMC10223123

[B8] Fan Q. Xie Z. Zhao J. Hua J. Wei Y. Li X. . (2024). Simultaneous differential detection of H5, H7 and H9 subtypes of avian influenza viruses by a triplex fluorescence loop-mediated isothermal amplification assay. Front. Vet. Sci. 11, 1419312. doi: 10.3389/fvets.2024.1419312 39015104 PMC11250583

[B9] Fusaro A. Pu J. Zhou Y. Lu L. Tassoni L. Lan Y. . (2024). Proposal for a global classification and nomenclature system for a/H9 influenza viruses. Emerg. Infect. Dis. 30, e231176. doi: 10.3201/eid3008.231176 39043566 PMC11286050

[B10] Gu M. Xu L. Wang X. Liu X. (2017). Current situation of H9N2 subtype avian influenza in China. Vet. Res. 48, 49. doi: 10.1186/s13567-017-0453-2 28915920 PMC5603032

[B11] Guinat C. Tang H. Yang Q. Valenzuela Agüí C. Vaughan T. G. Scire J. . (2023). Bayesian phylodynamics reveals the transmission dynamics of avian influenza a(H7N9) virus at the human-live bird market interface in China. Proc. Natl. Acad. Sci. U.S.A. 120, e2215610120. doi: 10.1073/pnas.2215610120 37068240 PMC10151560

[B12] Hadfield J. Megill C. Bell S. M. Huddleston J. Potter B. Callender C. . (2018). Nextstrain: Real-time tracking of pathogen evolution. Bioinformatics 34, 4121–4123. doi: 10.1093/bioinformatics/bty407 29790939 PMC6247931

[B13] Hassan M. Z. Sturm-Ramirez K. Islam M. S. Afreen S. Rahman M. Z. Kafi M. A. H. . (2023). Interpretation of molecular detection of avian influenza a virus in respiratory specimens collected from live bird market workers in dhaka, Bangladesh: Infection or contamination? Int. J. Infect. Dis. 136, 22–28. doi: 10.1016/j.ijid.2023.08.020 37652093 PMC11849796

[B14] Jakob C. Paul-Stansilaus R. Schwemmle M. Marquet R. Bolte H. (2022). The influenza a virus genome packaging network — complex, flexible and yet unsolved. Nucleic Acids Res. 50, 9023–9038. doi: 10.1093/nar/gkac688 35993811 PMC9458418

[B15] Kang M. Lau E. H. Y. Guan W. Yang Y. Song T. Cowling B. J. . (2017). Epidemiology of human infections with highly pathogenic avian influenza a(H7N9) virus in guangdong 2016 to 2017. Euro Surveill 22, 30568. doi: 10.2807/1560-7917.ES.2017.22.27.30568 28703705 PMC5508330

[B16] Lemey P. Rambaut A. Drummond A. J. Suchard M. A. (2009). Bayesian phylogeography finds its roots. PloS Comput. Biol. 5, e1000520. doi: 10.1371/journal.pcbi.1000520 19779555 PMC2740835

[B17] Li C. Chen H. (2021). H7N9 influenza virus in China. Cold Spring Harb. Perspect. Med. 11, a038349. doi: 10.1101/cshperspect.a038349 32205415 PMC8327827

[B18] Li S. Cao J. Yan Y. Deng W. He Y. Xiang S. . (2026). Longitudinal wastewater-based epidemiology reveals the spatiotemporal dynamics and genotype diversity of diarrheal viruses in urban guangdong, China. Viruses 18, 83. doi: 10.3390/v18010083 41600847 PMC12846444

[B19] Li X. Patel V. Duan L. Mikuliak J. Basran J. Osgood N. D. (2024). Real-time epidemiology and acute care need monitoring and forecasting for COVID-19 via bayesian sequential monte carlo-leveraged transmission models. Int. J. Environ. Res. Public Health 21, 193. doi: 10.3390/ijerph21020193 38397684 PMC10888645

[B20] Liu S. Zhuang Q. Wang S. Jiang W. Jin J. Peng C. . (2020). Control of avian influenza in China: Strategies and lessons. Transbound Emerg. Dis. 67, 1463–1471. doi: 10.1111/tbed.13515 32065513

[B21] Lu J. Raghwani J. Pryce R. Bowden T. A. Thézé J. Huang S. . (2018). Molecular evolution, diversity, and adaptation of influenza a(H7N9) viruses in China. Emerg. Infect. Dis. 24, 1795–1805. doi: 10.3201/eid2410.171063 30226157 PMC6154164

[B22] Naguib M. M. Li R. Ling J. Grace D. Nguyen-Viet H. Lindahl J. F. (2021). Live and wet markets: Food access versus the risk of disease emergence. Trends Microbiol. 29, 573–581. doi: 10.1016/j.tim.2021.02.007 33712334 PMC9189808

[B23] Sharma S. P. Chawla-Sarkar M. Sandhir R. Dutta D. (2024). Decoding the role of RNA sequences and their interactions in influenza a virus infection and adaptation. Wiley Interdiscip. Rev. RNA 15, e1871. doi: 10.1002/wrna.1871 39501458

[B24] Sreenivasan C. C. Li F. Wang D. (2024). Emerging threats of highly pathogenic avian influenza a (H5N1) in US dairy cattle: Understanding cross-species transmission dynamics in mammalian hosts. Viruses 16, 1703. doi: 10.3390/v16111703 39599818 PMC11598956

[B25] Su W. Sia S. F. Choy K.-T. Ji Y. Chen D. Lau E. H. Y. . (2021). Limited onward transmission potential of reassortment genotypes from chickens co-infected with H9N2 and H7N9 avian influenza viruses. Emerging Microbes Infections 10, 2030–2041. doi: 10.1080/22221751.2021.1996209 34666614 PMC8567909

[B26] Sun Z. Li Y.-P. An Q. Gao X. Wang H.-B. (2023). Risk factors contributing to highly pathogenic avian influenza H5N6 in China 2014-2021: Based on a MaxEnt model. Transbound Emerg. Dis. 2023, 6449392. doi: 10.1155/2023/6449392 40303723 PMC12017038

[B27] Tian J. Bai X. Li M. Zeng X. Xu J. Li P. . (2023). Highly pathogenic avian influenza virus (H5N1) clade 2.3.4.4b introduced by wild birds, Chin. Emerg. Infect. Dis. 29, 1367–1375. doi: 10.3201/eid2907.221149 37347504 PMC10310395

[B28] Tisza M. Javornik Cregeen S. Avadhanula V. Zhang P. Ayvaz T. Feliz K. . (2023). Wastewater sequencing reveals community and variant dynamics of the collective human virome. Nat. Commun. 14, 6878. doi: 10.1038/s41467-023-42064-1 37898601 PMC10613200

[B29] Vale F. F. Vítor J. M. B. Marques A. T. Azevedo-Pereira J. M. Anes E. Goncalves J. (2021). Origin, phylogeny, variability and epitope conservation of SARS-CoV-2 worldwide. Virus Res. 304, 198526. doi: 10.1016/j.virusres.2021.198526 34339772 PMC8323504

[B30] Wang Z. Li H. Li Y. Wu Z. Ai H. Zhang M. . (2023). Mixed selling of different poultry species facilitates emergence of public-health-threating avian influenza viruses. Emerg. Microbes Infect. 12, 2214255. doi: 10.1080/22221751.2023.2214255 37191631 PMC10215028

[B31] Xue R. Ma H. Jiang Z. Xing L. Wang G. Lan Z. . (2025). Diversity of the H9N2 avian influenza virus in shandong province, China. Transboundary Emerging Dis. 2025, 1432483. doi: 10.1155/tbed/1432483 40302737 PMC12016696

[B32] Yuan R. Zou L. Kang Y. Wu J. Zeng X. Lu J. . (2016). Reassortment of avian influenza a/H6N6 viruses from live poultry markets in guangdong, China. Front. Microbiol. 7, 65. doi: 10.3389/fmicb.2016.00065 26903958 PMC4742543

[B33] Zhu B. Fung K. Feng H. H. Beatty J. A. Hill F. Tse A. C. N. . (2025). The hemagglutinin proteins of clades 1 and 2.3.4.4b H5N1 highly pathogenic avian influenza viruses exhibit comparable attachment patterns to avian and mammalian tissues. J. Virol. 99, e0097625. doi: 10.1128/jvi.00976-25 40985723 PMC12548461

