# Supplementary material for: A cross-sectional study of avian influenza virus in poultry markets of Guangdong China, 2025
Source: Front Cell Infect Microbiol. 2026 Aug 11;16:1914072. doi: 10.3389/fcimb.2026.1914072 (PMC13503173; doi:10.3389/fcimb.2026.1914072)
Supplement: Supplementary file 1 [file Table1.docx]

Supplementary Material

**Supplementary Figure 1.** **Relative abundance of AIV neuraminidase subtypes in wastewater samples from Guangdong live poultry markets, May 2025.** The stacked bar chart displays the percentage composition of NA subtypes identified by targeted next-generation sequencing in wastewater samples collected from the representative cities. The number of samples (n) for each city is indicated on the x-axis. The dominant NA subtypes varied by region, with N2 being most prevalent in Dongguan and Meizhou, and N6 in Qingyuan. This analysis is complementary to the hemagglutinin data presented in Figure 3B.

**Supplementary Figure 2. Time-scaled phylogenetic analysis of the hemagglutinin genes of AIVs identified in live poultry markets of Guangdong Province.** Maximum likelihood (ML) time-scaled phylogenetic trees were constructed for the HA genes of (**A**) H5, (**B**) H7, (**C**) H9, and (**D**) H6 subtypes, illustrating the temporal evolutionary dynamics and divergence times of the AIV HA gene sequences obtained in this study.
